# Income level and regional policies, underlying factors associated with unwarranted variations in conservative breast cancer surgery in Spain

**DOI:** 10.1186/1471-2407-11-145

**Published:** 2011-04-19

**Authors:** Manuel Ridao-López, Sandra García-Armesto, Begoña Abadía-Taira, Salvador Peiró-Moreno, Enrique Bernal-Delgado

**Affiliations:** 1 Instituto Aragonés de Ciencias de la Salud. Instituto de Investigación Sanitaria Aragón. Zaragoza, Spain; 2 Centro Superior de Investigación en Salud Pública.(CSISP) Valencia, Spain

## Abstract

**Background:**

Geographical variations in medical practice are expected to be small when the evidence about the effectiveness and safety of a particular technology is abundant. This would be the case of the prescription of conservative surgery in breast cancer patients. In these cases, when variation is larger than expected by need, socioeconomic factors have been argued as an explanation. Objectives: Using an ecologic design, our study aims at describing the variability in the use of surgical conservative versus non-conservative treatment. Additionally, it seeks to establish whether the socioeconomic status of the healthcare area influences the use of one or the other technique.

**Methods:**

81,868 mastectomies performed between 2002 and 2006 in 180 healthcare areas were studied. Standardized utilization rates of breast cancer conservative (CS) and non-conservative (NCS) procedures were estimated as well as the variation among areas, using small area statistics. Concentration curves and dominance tests were estimated to determine the impact of income and instruction levels in the healthcare area on surgery rates. Multilevel analyses were performed to determine the influence of regional policies.

**Results:**

Variation in the use of CS was massive (4-fold factor between the highest and the lowest rate) and larger than in the case of NCS (2-fold), whichever the age group. Healthcare areas with higher economic and instruction levels showed highest rates of CS, regardless of the age group, while areas with lower economic and educational levels yielded higher rates of NCS interventions. Living in a particular Autonomous Community (AC), explained a substantial part of the CS residual variance (up to a 60.5% in women 50 to 70).

**Conclusion:**

The place where a woman lives -income level and regional policies- explain the unexpectedly high variation found in utilization rates of conservative breast cancer surgery.

## Background

Breast cancer represents 30% of all cancers in Spain and is the most common tumor in women, responsible for very high morbidity and mortality rates. About 16,000 cases are diagnosed annually in the country representing a death burden of almost 6,000 women [1]. Early detection programs along with diagnostic and therapeutic advances have raised survival rates five years after diagnosis over 75% [2].

The current therapeutic approach for breast cancer includes surgery, followed by hormonal therapy and radiotherapy. Surgical treatment can be conservative (CS), which preserves part of breast glandular tissue, or non-conservative treatment (NCS) which entails total removal of breast glandular tissue, maintaining or not the skin tissue.

Different studies [3-6] show equal effectiveness for both surgical strategies in terms of long-term survival. In fact CS is recommended, at any stage of breast cancer [5,6], confining the use of NCS to those situations where the tumor's size relative to total breast mass prevents conservative resection.

In spite of these recommendations, some studies have pointed out differences in the use of surgical techniques [7-11]. Socioeconomic factors such as poverty, educational level, urbanity and race, showed strong association to the use of CS. However, the analysis of this variability and its underlying factors is still pending in the Spanish context.

Our work therefore aims to describe the variability in the use of conservative surgery versus the non-conservative option in patients with breast cancer in Spain; the goal is to establish whether there is any relationship between the techniques used and health care areas' socioeconomic level, ruling out alternative explanations.

## Methods

### Design

Ecologic descriptive study of geographic variations in the rates of CS versus NCS in breast cancer, and the association between them and the socioeconomic level of the health care areas.

### Population and setting

Object of analysis were 180 health care areas of 16 Autonomous Communities (ACs) participating in the project Atlas of Variations in Medical Practice in the National Health System [12]. These 180 areas comprised a female population in annual average between 2002 and 2006 of 16,269,475 women over 15 years old.

We selected all discharges with female breast cancer recorded in the Hospital Discharge Administrative Database (HDAD) between the years 2002 to 2006. Of these, we selected those episodes in which breast surgery had been performed. Cases were classified according to whether they had received CS or NCS. In order to achieve consistency in the operational definitions a group of surgeons expert in breast surgery, sorted the corresponding ICD 9^th ^MC surgical procedures codes into CS and NCS. Table 1 shows the resulting operational definitions.

**Table 1:** Breast cancer surgery ICD 9th codes and definitions

Breast cancer	174 Malignant neoplasm of female breast
	174.0 Nipple and areola
	174.1 Central portion
	174.2 Upper-inner quadrant
	174.3 Lower-inner quadrant
	174.4 Upper-outer quadrant
	174.5 Lower-outer quadrant
	174.6 Axillary tail
	174.8 Other specified sites of female breast
	174.9 Breast (female), unspecified
	233.0 Carcinoma in situ breast
	V10.3 Breast. History of conditions classifiable to 174 and 175
**Conservative mastectomy**	85.20 Excision or destruction of breast tissue, not otherwise specified
	85.21 Local excision of lesion of breast
	85.22 Resection of quadrant of breast
	85.23 Subtotal mastectomy

**Non conservative mastectomy**	85.33 Unilateral subcutaneous mammectomy with synchronous implant
	85.34 Other unilateral subcutaneous mammectomy
	85.35 Bilateral subcutaneous mammectomy with synchronous implant
	85.36 Other bilateral subcutaneous mammectomy
	85.41 Unilateral simple mastectomy
	85.42 Bilateral simple mastectomy
	85.43 Unilateral extended simple mastectomy
	85.44 Bilateral extended simple mastectomy
	85.45 Unilateral radical mastectomy
	85.46 Bilateral radical mastectomy
	85.47 Unilateral extended radical mastectomy
	85.48 Bilateral extended radical mastectomy

Being a geographical study (examining population rather than individual exposure to procedures), breast cancer interventions were assigned to the patient's geographic area of residence regardless of the hospital where they were operated. These cases constituted the numerator of the rates and the female population in each area was the denominator [12].

### Main endpoints

Standardized CS and NCS rates for women over 15 years old were calculated as well as the specific rates in three age groups (from 15 to 49, 50 to 69 and 70 and over). The intermediate age-group was chosen to represent the women targeted for population breast cancer screening in Spain (50 to 70 years). The age standardization was performed using the direct method, applying as reference the female population registered in the 2003 census for all the health areas.

### Independent socioeconomic variables

1) The "economic level" is understood as an index of average family income available per capita (income from work, capital income, social benefits and transfers, minus direct taxes paid by families and the fees paid to social security). This variable was stratified into 10 categories (1: <7,200 Euros; 2: 7,200 to 8,300; 3: 8,300 to 9,300; 4: 9,300 to 10,200; 5: 10,200 to 11,300; 6: 11,300 to 12,100; 7: 12,100 to 12,700; 8: 12,700 to 13,500; 9: 13,500 to 14,500 and 10:> 14,500)

2) The "educational level" is understood as the percentage of college graduates within the total population.

Spain municipalities' Economic Yearbook 2005 [13] was the source of up to date data for economic level (2003) and educational level (2001). Each area's economic and educational levels were calculated as population weighted averages of the values registered for each municipality included in a healthcare area.

### Alternative explanatory factors

1) Availability of high technology: living in an area served by a tertiary hospital or by a hospital delivering radiotherapy services could have an impact on the election of conservative vs non-conservative surgery; the absence of these services in the neighbouring facilities would entail regularly travelling to the closest centre providing adjuvant therapy which might be bothersome; that could be specially true in elder women [11]. Hypothetically, those areas served by "high-tech" hospitals should show higher rates of conservative (vs non-conservative) surgery. The 2003 National Survey of Inpatient Care Premises (ESCRI) [14] provided the technology availability data needed to study this potential association.

2) "Autonomous community of residence" will be considered as a surrogate of regional policies (i.e. breast cancer screening).

Due to the fact that the Spanish National Health System is organized in ACs with full responsibility on planning and implementation of specific intervention programs [e.g., breast cancer screening], it is expected a cluster effect explaining part of the variation on surgery rates.

### Analysis

To determine the magnitude of the systematic variation between rates (beyond chance and differences in population structure), the usual small area analysis statistics were calculated [12]: ratio of variation (RV), systematic component of variation (SCV) and Empirical Bayes statistic (EB) [15]. In the case of RV, in order to reduce random noise, only the areas between 5 and 95 percentile of the corresponding rates distribution were used for the estimation.

The association between intervention rates and the socio-economic level (economic and educational level) of the area, was established calculating the concentration index and graphically represented drawing concentration curves. Likewise, the level of dominance of each concentration curve was determined by comparing it to the diagonal line of 45°. The concentration curve shows the surgery rates (CS and NCS) ordered by area income (growing). A concentration curve falling below the diagonal (concentration index >0) indicates that surgery rates are less concentrated in the first population group (lower quintiles of economic level) and more concentrated in the last population groups (higher quintiles of economic level). Conversely, a concentration curve located above the diagonal (concentration index < 0) indicates that surgical rates are more concentrated in the first population groups (lower economic level) and less concentrated in the last population groups (higher economic level) [16,17].

The effects of high technology availability and regional policies, were studied using ANOVA analysis (bivariate analysis) followed by mixed-effects linear multilevel hierarchical models. The best model was selected on the basis of the likelihood ratio with a 5% threshold on alpha error. Rho statistic and its confidence interval were estimated to determine the amount of variation explained by the second level (ACs) once first level variables (demography, socioeconomic status, supply factors) were adjusted [18,19].

All analyses were performed using STATA 10 [19] and DASP [20] software.

### Ethics

The study, observational in design, uses retrospective data from administrative databases in where patients are anonymous to the researchers. The study did not require informed consent nor Ethics Committee approval.

## Results

A total of 81,868 breast cancer interventions were performed across all the age groups: 44,648 were CS and 38,067 NCS. The corresponding standardized rates amounted to 5.29 and 4.84 interventions for every 10,000 women; this implies an overall 9.3% more utilization of conservative surgery.

**Figure 1:**
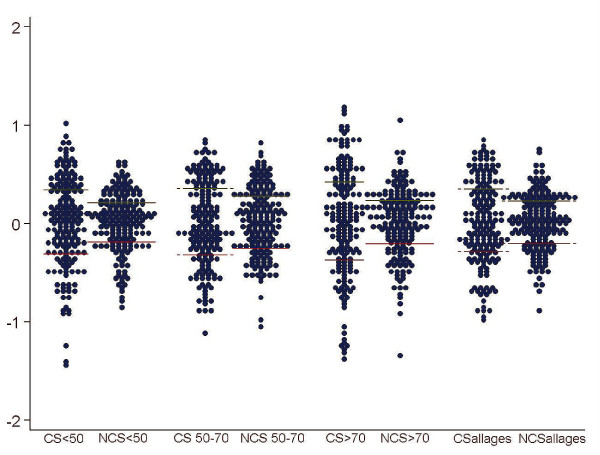
**Variation in conservative vs non conservative breast cancer surgical rates 2002 - 2006 log standardized rates by age group**. CS: conservative surgery; NCS: Non-conservative surgery. Each dot represents a healthcare area standardized rate per 10,000 women.

Figure 1 and Table 2 show the variability in the intervention rates across healthcare areas. The statistics detect large variability in utilization of CS: in terms of variation, the areas in the upper side of the distribution perform 4 times more conservative interventions than those in the lower end; in turn, NCS shows more moderate levels of variability with a ratio around 2 between the areas with highest and lowest utilization; in SCV terms, systematic variation (beyond random) was 1.91 times higher in the case of conservative surgery compared to non-conservative interventions.

**Table 2:** Conservative and non conservative breast cancer surgical rates

	Less than 50 years			50 to 70 years			More than 70 years			All ages		
	
	CS	NCS	Total	CS	NCS	Total	CS	NCS	Total	CS	NCS	Total
**Cases**	11,821	9,646	21,251	23,204	14,676	35,523	9,623	13,745	23,094	44,648	38,067	81,868
**Population**	47,728,827			19,931,718			13,945,734			81,606,279		
**Standardized rate**	2.40	2.05	4.41	11.13	7.69	18.67	6.81	10.30	16.91	5.29	4.84	10.03

**RV**_**5-95**_	4.17	2.76	2.45	3.97	2.98	2.32	7.16	2.909	2.50	3.92	2.51	2.05
**SCV**	0.141	0.042	0.046	0.148	0.108	0.062	0.236	0.122	0.083	0.153	0.080	0.057
**Empirical Bayes**	0.149	0.064	0.048	0.182	0.101	0.061	0.272	0.091	0.067	0.191	0.078	0.059

2002-2006 age-sex standardized rates and statistics of variation

CS: Conservative surgery; NCS: Non-conservative Surgery. RV_5-95: _Represents the range of variation in standardized rates, between the areas in percentiles 95^th ^and 5^th^, expressed as a ratio. Systematic Component of Variation (SCV)) and Empirical Bayes Statistic (EB) represent the variation considered systematic (thus, beyond chance). Both are estimated using observed and expected cases; the latter obtained by indirect standardization.

EB uses Bayesian modelling and simulation, resulting in a more robust estimate for small numbers [15].

The analysis by age group quite follows the same trend depicted by the overall figures. CS is more frequently used (always showing higher variation across healthcare areas than NCS) except in the group 70 years and older; among elder women the NCS utilization rate is clearly above the CS (table 2). In SCV terms, systematic variation is larger in conservative surgery; particularly, in the age group below 50 years old, showing a 3.3 times higher systematic variation in conservative techniques (1.37 higher for women between 50 and 70 years old and 1.9 for women above 70).

Applying the Empirical Bayes analysis (more robust statistics), systematic variation in CS is still higher than in NCS (between 1.8 to 3 times higher). In general, the pattern of Bayesian statistics was consistent with the results of the initial analysis, with the two types of surgery showing the same behavior across age groups, and consistently different between themselves.

**Figure 2:**
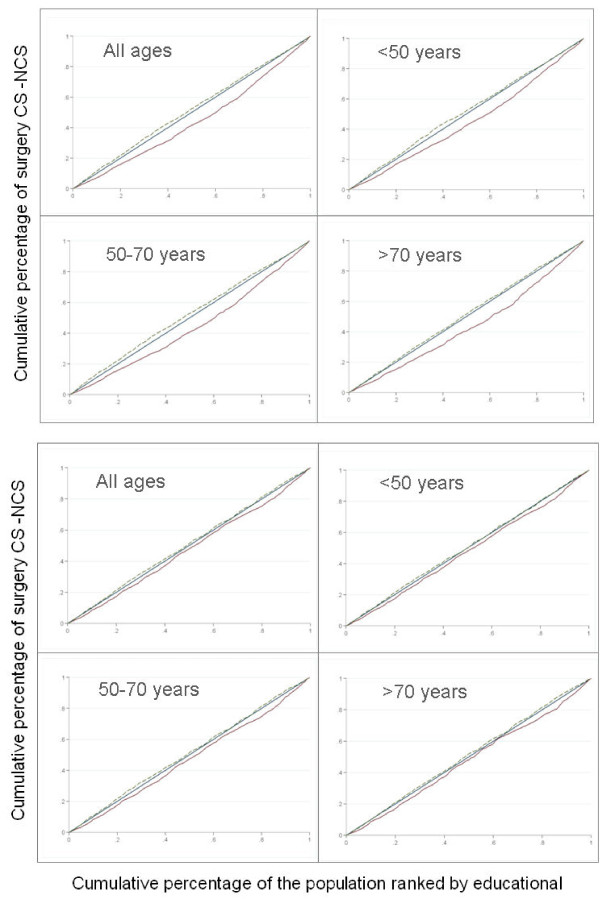
**Concentration curves for conservative and non conservative surgery by age-group, area income and area educational level**. X axis represents quintiles of the distribution of socioeconomic level; y axis represents quintiles of the distribution of rates of surgery. Solid line represents conservative surgery. Dashed line represents non conservative surgery. Explanation: the level of dominance of each concentration curve is determined by comparing it to the diagonal line of 45°. If the concentration curve is below the diagonal (concentration index >0), it is indication that surgery rates are less concentrated in the first population group (lower economic level) and more concentrated in the last population groups (higher economic level). If the concentration curve is located above the diagonal (concentration index < 0) it indicates that surgical rates are more concentrated in the first population groups (lower economic level) and less concentrated in the last population groups (higher economic level).

As for the association between socio-economic variables and types of surgical interventions, the concentration index observed for the NCS was systematically negative, while systematically positive for CS (table 3 and figure 2). These results mean that those areas with higher economic and educational levels show higher rates of CS, regardless of the age group, while areas with lower economic and educational levels showed higher rates of NCS interventions. These differences in socio-economic variables were statistically significant when compared to line 45° only in the case of CS. Particularly, the economic level was significant in analyzing any age group; in turn, the educational variable proved significant when analyzing the total population and the medium-age group (50 to 70 years old) but not for the younger and elder women. [Additional file 1]

**Table 3:** Concentration index values for conservative and non conservative surgery

Age-groups	Concentration Index ranked by income (CI95%)	Concentration Index ranked by educational level (CI95%)
All years CS	0.12 (0.09 to 0.14) ‡	0.05 (0.01 to 0.09) ‡
All years NCS	-0.03 (-0.05 to -0.007) ‡	-0.02 (-0.04 to 0.001)
< 50 years CS	0.10 (0.07 to 0.14) ‡	0.04 (0.01 to 0.08) ‡
< 50 years NCS	-0.02 (-0.05 to -0.002) ‡	-0.01 (-0.03 to 0.01)
50-70 years CS	0.12 (0.09 to 0.15) ‡	0.05 (0.01 to 0.10) ‡
50-70 years NCS	-0.04 (-0.06 to -0.01) ‡	-0.02 (-0.05 to 0.01)
> 70 years CS	0.13 (0.09 to 0.17) ‡	0.04 (-0.02 to 0.11)
> 70 years NCS	-0.02 (-0.04 to 0.006)	-0.02 (-0.05 to 0.007)

CS: Conservative Surgery; NCS: Non-conservative surgery. CI95%: Concentration index confidence interval assuming a type I error of 5%. ‡ Statistical significance

With regard to the effect of living in an area served by "high-tech" hospitals, neither the existence of a tertiary centre nor the availability of radiotherapy services within the area had any statistically significant effect on differences in surgery rates. Only a slightly higher rate of CS was observed in the 50 to 70 age group in areas served with radiotherapy (12 vs 10.8 per 10,000 women); in turn, a slightly higher rate of NCS was found in the elder group, in those areas without radiotherapy service (10.6 vs 9.5 per 10,000 women). [Additional file 1]

The multilevel analysis for CS discarded the educational level variable and only economic level was retained in the model. When the cluster effect of the autonomous community was incorporated into the analysis, it accounted for 34.7% of the residual variation for all women (Confidence Interval, CI, 95%: 16.9%-58.2%). Analysing by age groups, it can be observed how AC accounted for up to 23% of residual variation in the case of women below 50 years old (CI95%: 6.8% to 55.2%), 36.7% in the case of women between 50 and 70 years old (CI95%: 18.1% to 60.5%) and 25.9% in the case of women older than 70 years (CI95%: 13.6% to 43.7%). Income availability in the area and the AC of residency did not show interaction. [Additional file 2]

As for the NCS multilevel analysis, neither educational nor economic variables remained in the model, confirming the expectation suggested by the results of the bivariate analysis. In this case the AC (second level) barely explained a 3.5% of the variation in all women (CI95%: 0.06% to 65.5%). In turn, it explained a small part of the residual variation in the youngest group of age, 14% (CI95%: 4.5% to 35.7%), up to a 4.9% (CI95%: 0.5% to 34.8%) in the case of women between 50 and 70 years old, and 1% of residual variation (CI95%: 0.001% to 87%) in the case of women above 70 years old. [Additional file 2]

## Discussion

This ecological study on CS vs. NCS utilization, in 180 Spanish healthcare areas, has shown that: a) there are systematic variations in the use of these surgical procedures that are not amenable to population age structure; b) The use of CS is growing but in an uneven fashion incongruent with its being an effective surgical treatment for breast cancer; utilisation rates are highly dependent upon economic level in the area regardless the age group analyzed; affluent areas show consistently higher CS rates while poorer areas yield consistently higher NCS rates; c) supply of high technology (radiotherapy services within a healthcare area) was not related to differences in CS or NCS rates; and, d) Regional policy (i.e. breast cancer screening programs) behaves as an independent factor in explaining the use of one or another surgical procedure.

A good part of the detected variation in the use of CS was explained by economic level and regional policy (AC); this construct seems to be especially effective in explaining CS variations for women aged 50 to 70. In the case of NCS, the AC explained a small part of the variance.

### Consistency with previous knowledge

These results show some level of incongruence, with what was expected for this type of procedure. According to the uncertainty hypothesis formulated to explain geographical variations of medical practice [21], effective and safe procedures should have low variability, lower than those procedures in which the risk-benefit balance is less certain. There is a high level of consensus supporting the indication of surgery for diagnosed breast cancer. Recent evidence points out how in Spain the variability in the use of mastectomy is among the lowest for cancer related procedures [22]. Focusing on the two options (conservative and non-conservative), the evidence-based recommendation for the use of CS prevails over NCS, the size of the tumor being the only limiting factor. However, a higher variation in the use of CS has been observed compared to NCS; this holds even in cases where variation was completely unexpected, such as the age group between 50 and 70 years; being the targeted population for existing screening programs, these women have a higher probability of early detection and thus smaller size at diagnosis.

On the other hand, regarding the effect of socio-economic variables, the results of this study are consistent with other studies. Gilligan [7] highlights, how 10 years after the publication of the first trials on CS-NCS, socio-demographic factors, such as per capita income levels and education, still influence the use of CS versus NCS and thus women who reside in more deprived areas have a higher probability of not receiving CS. The authors affirm that the explanation should not be sought in differing levels of medical knowledge but rather in different levels of access to medical care. In addition, Smith [10] detects considerable geographic variations in the use of CS notwithstanding the overall increasing tendency to use CS instead of NCS in the USA; according to their results, 70% of the women susceptible of mastectomy were treated with CS in the Northeast region of USA (wealthier areas) against a 50% in the south of the USA (less affluent areas).

Finally, the observed AC cluster effect is congruent with the role of ACs in planning and implementing screening policy. Existing evidence on breast cancer screening programs across Spain points out the differences on degree of implementation, design and coverage for the period of analysis [23]. Such differences could be argued as relevant in explaining the detected regional effect: effective early detection translates into smaller size of the tumor, thus discarding the primary indication for NCS. In Spain the target population for screening is women between 50 and 70 years old; therefore, the impact of the regional factor in this age group can be expected to be relevant. In fact, AC of residence explained up to a 60% of the residual variation in this age-group.

Unfortunately comparable data about the characteristics of each of those regional screening programs are scarce. A thorough analysis of the characteristics of the regional breast cancer screening programs [24] concluded that the information systems supporting them were too heterogeneous to allow for sounded comparisons across ACs; as the authors plead, only standardized definitions and enhanced systematic data collection would enable the benchmarking of the relevant aspects of screening programs (both results and strategy).

Nevertheless, in an attempt to test the suggested hypothesis an exploratory analysis was conducted using two of the indicators collected in the aforementioned study: adherence to the screening program among targeted women and rate of T1 tumors detected [24]. The analysis showed how those regions achieving higher adherence among targeted women registered higher rates of CS (Spearmann coefficient = 0.52; p = 0.04). In turn, and more specifically, the higher the effectiveness of a regional screening program in detecting T1 tumors, the higher the CS rates registered in the AC (Spearmann coefficient = 0.87; p < 0.001). Though revealing, given the weakness of the data, no conclusions can be drawn from these findings on the precise nature of the effect of regional policy on the variability in mastectomy modality and further analysis is warranted.

### Alternative explanations for the obtained model

Several factors besides age, population income and AC of residence should be considered as an alternative explanation to the CS rates variability. The following paragraphs will discuss whether the impact of these factors might alternatively explain our findings.

#### Do differences in tumors size explain the differences in rates?

According to evidence the key factor in deciding the use of CS is the tumor size [6,11,25]. Arguably high CS rates might not depend upon age or high income, but on higher proportions of smaller tumors in those areas with higher CS rates. Unfortunately this information item (i.e. tumor status "T") is not available in hospital administrative databases or in cancer population-based regional registries across the country. Therefore differences in the T1-T4 case-mix among areas are unknown.

However, we could reasonably argue that since the prevalence of small tumors is highly dependant on the screening effectiveness (therefore regional policies), the lack of data on tumor size and the eventual differences in T1-T4 case-mix across areas are dealt with once autonomous community cluster effect is included in the models.

#### Are there differences across healthcare areas regarding the speed of adoption of conservative surgery technology?

Due to the nature of the study, a cross-sectional study, another alternative explanation for the observed variation in utilization rates relies on eventual differences in the speed of adoption of CS techniques across healthcare areas. After examining the evolution in utilization between 2002 and 2006, we observed an overall increasing trend in the use of CS compared to NCS, more pronounced for the group of women aged 50 to 70 years. The *ad hoc *joint point analysis performed [26,27] confirmed a statistically significant trend change in 2004 favoring the utilization of CS [additional file 3].

Nevertheless, a concurrent study [28] looking specifically at the adoption patterns of CS in two of the Spanish Autonomous Communities (including 40% of healthcare areas of our work) showed no differential paces in the adoption of CS techniques between the two ACs, minimizing the possibility of speed of adoption as an alternative explanation.

An additional argument for discarding differential speed of adoption comes hand by hand with the results obtained in analyzing the impact of proximity to tertiary hospitals and radiotherapy premises. CS adoption is closely related to innovative environments; the high tech supply factors included in the analysis can be reasonably considered as surrogate variables for adoption speed. Since, none of these supply variables showed any relationship with CS rates, we do not expect adoption speed to provide an alternative explanation to the variability detected.

#### Unaccounted Factors

As noted, the adjusted models left a significant proportion of unexplained residual variation. Some factors not included in the analysis, may contribute to explain the unaccounted variance. The availability of plastic surgeons during surgery, local learning cascades [29,30], recommendations by leading surgeons and oncologists [31-33] and patients' differential preferences (particularly for women under 50 and older than 70 years) [34,35] have been highlighted as relevant factors in the literature. The eventual different geographical distribution of any of these factors may play a role in explaining the detected variation in utilisation rates across healthcare areas.

The supply-related set of factors (availability of plastic surgeons or learning cascades) can be logically clustered together into "innovativeness of the environment". As argued in the case of speed of innovation adoption, no association has been detected between living in an area served by a "high tech centre" and the risk of receiving either type of surgery.

Regarding the last factor it is worth noting that patients' preferences are usually linked to education. Remarkably, population education level did not remain in the models, showing low power in explaining variation.

Summarizing: age, socioeconomic status and regional policies explain differences in the surgical approach at population level. Those potential alternative explanations like tumor size differences, speed in adoption of innovations and non studied supply and demand factors, seem to be controlled through the variables used as independent predictors of variation.

### Study Limitations

#### Design Type

Among the limitations of this study must be mentioned firstly, those inherent to its ecologic design: the detected socioeconomic gradient in intervention rates across healthcare areas prevents drawing conclusions about individual access to healthcare driven by their economic levels (ecological fallacy). However, as noted earlier, individual-based studies reached the same conclusions regarding the socioeconomic impact on CS rates.

#### Selection bias

The study only included data from hospitals within the public utilization network. If private hospitals were to perform more CS, the inclusion of private hospitals would have yielded different results as to the estimated intervention rates and the age and economic effects detected. The single study available in Spain comparing the use of CS vs NCS in public and private hospitals [28] refers to the AC of Catalonia where the private sector is stronger compared to the rest of the territory. They found lower CS use rates in private hospitals compared to public ones. At the light of these findings an eventual underestimation of CS utilization rates in more affluent areas (more privately served) is unlikely; in any case, the inclusion of private hospitals data would only reinforce the detected economical gradient.

Another potential source of selection bias in population studies comes from the coverage of the administrative data bases used. In theory, areas showing low levels of mastectomy could be denoting poor coverage in the registration of discharges rather than actual lower use of surgical procedures. However, the hospital discharges database used in this study registers above 95% of the actual discharges in all ACs [22].

A third possible source of error is linked to the success in assigning mastectomy cases to the place of residence (the base of the study is geographical and patients are analyzed according to their place of residence). Systematic variability in the proportion of successful assignment across geographical units would pose some doubts about underlying bias. Previous studies using this same cancer administrative data in Spain [22], showed assignment rates above 95% of the cases and homogeneous achievement across the territory. Therefore this source of error would not affect this study results.

#### Miss-classification of cases due to the ICD9th definitions

In using hospital discharges administrative databases (HDAD), cases are necessarily defined by ICD codes (in Spain ICD9-CM). This "language" do not allow for distinction between primary breast surgery, re-intervention in the same breast and intervention on the contralateral breast. This limitation creates a risk of double counting and case misclassification (e.g., initial CS followed by NCS due to recidive in the same breast or new CS for the contralateral breast). If misclassification were important, it would be necessary to consider whether it affects CS rates in a different manner across healthcare areas since the second intervention due to recidive will be less conservative. A Spanish study, in which the HDAD was compared with 4 population cancer registries, showed that, in the particular case of breast cancer (using the same definition applied for this study), the positive predictive value for the HDAD definition was above 80% [36]. The percentage of cases that could be considered as "misclassified", being recidive or contralateral breast interventions were only one in 100,000 people.

## Conclusion

As observed, living in a certain healthcare area or political demarcation of the country, affects the probabilities of a woman receiving one type of surgery or the other. In the case of CS, this probability is also influenced by the economic level of the population living there.

The revision of breast cancer screening programs' characteristics and the assessment of effective access to them in certain deprived areas is critical to reduce the detected underutilization of CS in certain places. In turn, smaller tumors once detected, will require timely availability of CS, whichever the socioeconomic status of the served population.

According to the literature women subject to NCS experience worse quality of life, physical image and satisfaction indicators than those undergoing CS [37-39]. Thus the women living in areas with higher NCS rates will presumably be in disadvantage to their peers living in areas with high CS rates were they to suffer breast cancer. This situation in areas with lower socioeconomic level must be tackled through strategies to increase effective early detection, particularly in a country where women have universal access to screening and treatment, without any copayment.

## Competing interests

The authors declare that they have no competing interests.

## Authors' contributions

MRL, SGA, MBAT, SPM and EBD have been involved in designing, discussing results, and in drafting the paper. MRL and EBD developed the analyses. All authors read and approved the final manuscript.

## Pre-publication history

The pre-publication history for this paper can be accessed here:

http://www.biomedcentral.com/1471-2407/11/145/prepub

## Supplementary Material

Additional file 1 **Age-standardized rates of breast cancer surgery and potential explanatory socioeconomic and supply factors, by type of intervention and age-group**. The file shows the different analyses aimed to explore the relationship between standardized-rates of mastectomy and, supply and demand factors potentially related to variation. Click here for file

Additional file 2 **Multilevel analyses modelling**. The file describes the most parsimonious models estimated to describe the factors affecting variation by type of intervention (conservative vs. non-conservative) and age-group. Click here for file

Additional file 3 **Joint point analysis of change in utilisation trends for conservative and non conservative surgery per age group**. The file illustrates the joint-model estimates, testing whether the early adoption of conservative surgery explained part of the variation. Click here for file
