# Supplementary material for: Income level and regional policies, underlying factors associated with unwarranted variations in conservative breast cancer surgery in Spain
Source: BMC Cancer. 2011 Apr 19;11:145. doi: 10.1186/1471-2407-11-145 (PMC3103476; doi:10.1186/1471-2407-11-145)
Supplement: Additional file 1 — Age-standardized rates of breast cancer surgery and potential explanatory socioeconomic and supply factors, by type of intervention and age-group. The file shows the different analyses aimed to explore the relationship between standardized-rates of mastectomy and, supply and demand factors potentially related to variation. [file 1471-2407-11-145-S1.DOC]

**Additional file 1**

**Age-standardized rates of breast cancer surgery and potential explanatory socioeconomic and supply factors, by type of interventions and age-group**

|  | **Variables** | **Quintile** | **Range of values *** | **All women**  **CS** | **All women**  **NCS** | **< 50** years  **CS** | **< 50** years  **NCS** | **50**to**70** years  **CS** | **50**to**70** years  **NCS** | **> 70** years  **CS** | **> 70** years **NCS** |
| --- | --- | --- | --- | --- | --- | --- | --- | --- | --- | --- | --- |
| **Socioeconomic factors** | Economic level | Q1  Q2  Q3  Q4  Q5 | <= 3.33  3.34- 4.27  4.28-5.77  5.78-7.15  >= 7.16 | ↑3.96  4.20  4.67  6.50  7.09 | 4.90  5.02  4.63  4.55  5.08 | ↑1.83  2.03  2.10  2.88  3.16 | 2.10  2.19  1.93  1.99  2.03 | ↑8.25  8.67  9.97  13.85  14.92 | 8.09  7.99  7.44  7.11  7.83 | ↑5.08  5.25  5.91  8.42  9.38 | 9.96  10.45  9.80  9.68  11.61 |
|  | Educational level | Q1  Q2  Q3  Q4  Q5 | <= 8.10  8.11- 9.50  9.51-11.20  11.21-14.20  >= 14.21 | ↑↓ 4.12  5.45  5.42  5.88  5.56 | 4.74  5.07  4.59  4.91  4.88 | ↑1.86  2.47  2.46  2.53  2.67 | 1.98  2.26  1.93  1.98  2.09 | ↑↓8.64  11.64  11.08  12.45  11.84 | 7.84  7.96  7.15  7.53  7.97 | ↑5.39  6.80  7.46  7.92  6.47 | 9.73  10.53  10.05  11.16  10.00 |
| **Supply Factors** | Beds per 1000 inhabitants | Q1  Q2  Q3  Q4  Q5 | <=1.47  1.48-1.81  1.82-2.25  2.26-2.92  >=2.93 | 5.09  4.91  4.98  5.33  6.12 | 4.63  4.39  5.11  5.02  5.03 | 2.27  2.31  2.30  2.38  2.74 | 1.99  1.96  2.04  2.10  2.15 | 10.62  10.22  10.55  11.50  12.76 | 7.44  6.84  8.22  8.11  7.84 | 6.81  6.20  6.22  6.62  8.18 | 9.64  9.18  11.25  10.57  10.89 |
|  | Physicians per 1000 inhabitants | Q1  Q2  Q3  Q4  Q5 | <=0.92  0.93-1.11  1.12-1.25  1.26-1.49  >=1.50 | 5.51  5.29  5.30  4.78  5.56 | 4.50  4.81  4.76  5.07  5.05 | 2.38  2.47  2.39  2.20  2.55 | 1.92  2.03  1.95  2.16  2.19 | 11.46  11.48  11.22  9.87  11.63 | 7.07  7.72  7.47  8.18  8.01 | 7.69  6.08  6.81  6.30  7.17 | 9.69  10.13  10.48  10.58  10.60 |
|  | Surgeons per 1000 inhabitants | Q1  Q2  Q3  Q4  Q5 | <=0.11  0.12-0.17  0.18-0.22  0.23-0.29  >=0.29 | 5.71  5.81  4.80  5.16  4.94 | ↑↓4.37  4.43  5.04  5.25  5.08 | 2.62  2.66  2.15  2.36  2.20 | 1.89  1.92  2.04  2.14  2.25 | 11.94  12.01  10.27  10.81  10.63 | ↑↓6.82  6.76  8.15  8.64  8.08 | 7.37  7.73  6.07  6.66  6.21 | 9.34  9.71  10.87  11.07  10.48 |
|  | MD under residency program per 1000 inhabitants | Q1  Q2  Q3  Q4  Q5 | <=0.00  0.01-0.10  0.11-0.20  0.21-0.33  >=0.34 | 5.32  5.08  4.73  5.98  5.30 | 5.06  5.01  4.90  4.80  4.40 | 2.24  2.47  2.17  2.79  2.35 | 2.02  2.05  2.02  2.14  2.01 | 11.17  10.59  10.30  12.38  11.17 | 8.08  7.85  7.78  7.85  6.86 | 7.49  6.15  5.55  7.74  7.01 | 11.13  11.07  10.67  9.57  9.04 |
|  | Tertiary hospital within the area | No  Yes |  | 5.19  5.57 | 4.92  4.59 | 2.36  2.51 | 2.04  2.08 | 10.94  11.72 | 7.86  7.15 | 6.66  7.27 | 10.55  9.53 |

Legend: Q: Quintile. Range of values: healthcare areas were distributed in quintiles with regard to their value within each factor; in the case of “tertiary hospital” factor which represents the existence of tertiary services within the healthcare area. The “range of values” column represents, for each factor, the range of values within a quintile. The remaining columns show the standardized-rates within each quintile. CS: Conservative Surgery; NCS: Non Conservative Surgery, MD: medical doctor. Arrows indicate statistically significant associations (p<0.05) and direction of association: ↑ direct trend; ↑↓: no trend.
