# Supplementary material for: Income level and regional policies, underlying factors associated with unwarranted variations in conservative breast cancer surgery in Spain
Source: BMC Cancer. 2011 Apr 19;11:145. doi: 10.1186/1471-2407-11-145 (PMC3103476; doi:10.1186/1471-2407-11-145)
Supplement: Additional file 2 — Multilevel analyses modelling. The file describes the most parsimonious models estimated to describe the factors affecting variation by type of intervention (conservative vs. non-conservative) and age-group. [file 1471-2407-11-145-S2.DOC]

**Additional file 2**

**Multilevel analysis modelling**

**2a Conservative surgery (all women)**

|  | **Model 1** | **Model 2 ‡** | **Model 3** |
| --- | --- | --- | --- |
|  | (area level variables) | (AC level added) | (allowing random slope) |
| *Fixed effects*  Standardized rate of surgery (*intercept (CI95%))*  *Area level variables*  *(beta coefficients (CI95%))*  Income level  Educational level  Tertiary hospital | 2.39 (1.40 to 3.37)  0.66 (0.52 to 0.82)  - 0.65 (-0.17 to 0.04)  0.44 (-0.27 to 1.18) | 3.36 (2.01 to 4.72)  0.31 (008 to 0.53)  -  - | 3.17 (1.75 to 4.59)  0.32 (0.05 to 0.60)  -  - |
| *Random effect*  *(variance (CI95%))*  Residual  Constant  Income level  Interaction (region*income)  Rho value (CI95%)  Likelihood ratio (p value) |  | 2.50 (2.01 to 3.12)  1.33 (0.41 to 4.35)  -  -  0.347 (0.169 to 0.582)  27.03 (0.0000) | 2.46 (1.97 to 3.08)  1.98 (0.13 to 30.8)  0.09 (0.01 to 1.01)  -0.32 (-1.39 to 0.74)  -  1.86 (0.3951) |

‡ Model chosen as the most parsimonious. Rho value represents the percentage of residual variation explained by the second level, in our case Autonomous Community (AC). Likelihood ratio evaluates the relative improvement of a model compared to the previous one. A p value below 0.05 would means

statistically significant improvement, and the model would be considered the best one explaining the variation in surgery rates. CI95%: Confidence Interval

with a type I error of 5%.

**2b Conservative surgery (women under 50**)

|  | **Model 1** | **Model 2 ‡** | **Model 3** |
| --- | --- | --- | --- |
|  | (area level variables) | (AC level added) | (allowing random slope) |
| *Fixed effects*  Standardized rate of surgery (*intercept (CI95%))*  *Area level variables*  *(beta coefficients (CI95%))*  Income level  Educational level  Tertiary hospital | 1.07 (0.58 to 1.57)  0.25 (0.17 to 0.33)  - 0.002 (-0.05 to 0.05)  0.09 (-0.28 to 0.45) | 1.45 (0.81 to 2.09)  0.16 (0.05 to 0.27)  -  - | 1.24 (0.42 to 2.05)  0.20 (0.04 to 0.36)  -  - |
| *Random effect*  *(variance (CI95%))*  Residual  Constant  Income level  Interaction (region*income)  Rho value (CI95%)  Likelihood ratio (p value) |  | 0.72 (0.57 to 0.90)  0.22 (0.04 to 1.11)  -  -  0.232 (0.068 to 0.552)  8.15 (0.0022) | 0.68 (0.55 to 0.85)  1.27 (0.29 to 5.59)  0.05 (0.01 to 0.20)  - 0.25 (-0.61 to 0.11)  6.07 (0.05) |

‡ Model chosen as the most parsimonious. Rho value represents the percentage of residual variation explained by the second level, in our case Autonomous Community (AC). Likelihood ratio evaluates the relative improvement of a model compared to the previous one. A p value below 0.05 would means

statistically significant improvement, and the model would be considered the best one explaining the variation in surgery rates. CI95%: Confidence Interval

with a type I error of 5%.

**2c Conservative surgery (women between 50 and 70**)

|  | **Model 1** | **Model 2 ‡** | **Model 3** |
| --- | --- | --- | --- |
|  | (area level variables) | (AC level added) | (allowing random slope) |
| *Fixed effects*  Standardized rate of surgery (*intercept (CI95%))*  *Area level variables*  *(beta coefficients (CI95%))*  Income level  Educational level  Tertiary hospital | 4.71 (2.60 to 6.83)  1.40 (1.07 to 1.73)  - 0.10 (-0.32 to 0.11)  0.80 (-0.75 to 2.36) | 7.21 (4.24 to 10.19)  0.62 (0.14 to 1.10)  -  - | 6.68 (3.64 to 9.72)  0.68 (0.09 to 1.28)  -  - |
| *Random effect*  *(variance (CI95%))*  Residual  Constant  Income level  Interaction (region*income)  Rho value (CI95%)  Likelihood ratio (p value) |  | 11.58 (9.27 to 14.46)  6.72 (2.04 to 22.11)  0.367 (0.181 to 0.605)  23.85 (0.000) | 11.46 (9.17 to 14.33)  8.43 (0.41 to 172.12)  0.36 (0.02 to 5.66)  -1.30 (-6.35 to 3.74)  1.48 (0.4780) |

‡ Model chosen as the most parsimonious. Rho value represents the percentage of residual variation explained by the second level, in our case Autonomous Community (AC). Likelihood ratio evaluates the relative improvement of a model compared to the previous one. A p value below 0.05 would means

statistically significant improvement, and the model would be considered the best one explaining the variation in surgery rates. CI95%: Confidence Interval

with a type I error of 5%.

**2d Conservative surgery (women over 70**)

|  | **Model 1** | **Model 2a** | **Model 2b ‡** | **Model 3** |
| --- | --- | --- | --- | --- |
|  | (area level variables) | (AC level added) | (AC level added) | (allowing random slope) |
| *Fixed effects*  Standardized rate of surgery (*intercept (CI95%))*  *Area level variables*  *(beta coefficients (CI95%))*  Income level  Educational level  Tertiary hospital | 3.55 (1.81 to 5.30)  1.04 (0.77 to 1.31)  -0.22 (-0.40 to -0.04)  1.18 (-0.10 to 2.47) | 3.63 (1.31 to 5.96)  0.42 (0.003 to 0.84)  0.04 (-0.11 to 0.19)  - | 3.76 (1.57 to 5.95)  0.48 (0.11 to 0.84)  -  - | 4.18 (2.41 to 5.94)  0.37 (-0.01 to 0.76)  -  - |
| *Random effect*  *(variance (CI95%))*  Residual  Constant  Income level  Interaction (region*income)  Rho value (CI95%)  Likelihood ratio (p value) |  | 7.89 (6.35 to 9.81)  2.94 (1.04 to 8.30)  0.271 (0.141 to 0.458)  31.89 (0.0000) | 0.259 (0.136 to 0.437)  34.91 (0.0000) | 7.89  0.003  0.08  -0.02  2.01 (0.3655) |

‡ Model chosen as the most parsimonious. Rho value represents the percentage of residual variation explained by the second level, in our case Autonomous Community (AC). Likelihood ratio evaluates the relative improvement of a model compared to the previous one. A p value below 0.05 would means statistically significant improvement, and the model would be considered the best one explaining the variation in surgery rates. CI95%: Confidence Interval with a type I error of 5%.

**2e Non-Conservative surgery (all women**)

|  | **Model 1** | **Model 2** |
| --- | --- | --- |
|  | (area level variables) | (AC level added) |
| *Fixed effects*  Standardized rate of surgery (*intercept (CI95%))*  *Area level variables*  *(beta coefficients (CI95%))*  Income level  Educational level  Tertiary hospital | 4.78 (4.02 to 5.54)  0.005 (-0.12 to 0.12)  0.01 (-0.06 to 0.09)  - 0.38 (-0.94 to 0.19) | 4.85 (4.59 to 5.11)  -  -  - |
| *Random effect*  *(variance (CI95%))*  Residual  Constant  Income level  Interaction (region*income)  Rho value (CI95%)  Likelihood ratio (p value) |  | 1.94 (1.54 to 2.42)  0.07 (0.001 to 4.61)  0.035 (0.0006 to 0.655)  0.27 (0.3031) |

‡ Model chosen as the most parsimonious. Rho value represents the percentage of residual variation explained by the

second level, in our case Autonomous Community (AC). Likelihood ratio evaluates the relative improvement of a model

compared to the previous one. A p value below 0.05 would means statistically significant improvement, and the model

would be considered the best one explaining the variation in surgery rates. CI95%: Confidence Interval with a type I

error of 5%.

**2f Non-Conservative surgery (women under 50**)

|  | **Model 1** | **Model 2** ‡ |
| --- | --- | --- |
|  | (area level variables) | (AC level added) |
| *Fixed effects*  Standardized rate of surgery (*intercept (CI95%))*  *Area level variables*  *(beta coefficients (CI95%))*  Income level  Educational level  Tertiary hospital | 2.13 (1.80 to 2.45)  -0.02 (-0.07 to 0.03)  0.003 (-0.03 to 0.04)  0.03 (-0.21 to 0.27) | 2.08 (1.93 to 2.23)  -  -  - |
| *Random effect*  *(variance (CI95%))*  Residual  Constant  Income level  Interaction (region*income)  Rho value (CI95%)  Likelihood ratio (p value) |  | 0.32 (0.26 to 0.40)  0.05 (0.01 to 0.22)  0.141 (0.045 to 0.357)  5.53 (0.0093) |

‡ Model chosen as the most parsimonious. Rho value represents the percentage of residual variation explained by the

second level, in our case Autonomous Community (AC). Likelihood ratio evaluates the relative improvement of a model

compared to the previous one. A p value below 0.05 would means statistically significant improvement, and the model

would be considered the best one explaining the variation in surgery rates. CI95%: Confidence Interval with a type I

error of 5%.

**2g Non-Conservative surgery (women between 50 and 70**)

|  | **Model 1** | **Model 2** |
| --- | --- | --- |
|  | (area level variables) | (AC level added) |
| *Fixed effects*  Standardized rate of surgery (*intercept (CI95%))*  *Area level variables*  *(beta coefficients (CI95%))*  Income level  Educational level  Tertiary hospital | 7.78 (6.31 to 9.24)  -0.12 (-0.35 to 0.10)  0.07 (-0.08 to 0.22)  -0.96 (-2.04 to 0.12) | 7.73 (7.20 to 8.26)  -  -  - |
| *Random effect*  *(variance (CI95%))*  Residual  Constant  Income level  Interaction (region*income)  Rho value (CI95%)  Likelihood ratio (p value) |  | 7.08 (5.70 to 8.81)  0.35 (0.03 to 4.55)  0.049 (0.005 to 0.346)  1.04 (0.1537) |

‡ Model chosen as the most parsimonious. Rho value represents the percentage of residual variation explained by the

second level, in our case Autonomous Community (AC). Likelihood ratio evaluates the relative improvement of a model

compared to the previous one. A p value below 0.05 would means statistically significant improvement, and the model

would be considered the best one explaining the variation in surgery rates. CI95%: Confidence Interval with a type I

error of 5%.

**2h Non-Conservative surgery (women over 70**)

|  | **Model 1** | **Model 2** |
| --- | --- | --- |
|  | (area level variables) | (AC level added) |
| *Fixed effects*  Standardized rate of surgery (*intercept (CI95%))*  *Area level variables*  *(beta coefficients (CI95%))*  Income level  Educational level  Tertiary hospital | 9.58 (7.51 to 11.65)  0.25 (-0.07 to 0.58)  - 0.03 (-0.25 to 0.18)  - 0.95 (-2.48 to 0.58) | 10.27 (9.65 to 10.88)  -  -  - |
| *Random effect*  *(variance (CI95%))*  Residual  Constant  Income level  Interaction (region*income)  Rho value (CI95%)  Likelihood ratio (p value) |  | 14.67 (11.84 to 18.18)  0.15 (0.0001 to 126.13)  0.01 (0.0001 to 0.874)  0.12 (0.3671) |

‡ Model chosen as the most parsimonious. Rho value represents the percentage of residual variation explained by the

second level, in our case Autonomous Community (AC). Likelihood ratio evaluates the relative improvement of a model

compared to the previous one. A p value below 0.05 would means statistically significant improvement, and the model

would be considered the best one explaining the variation in surgery rates. CI95%: Confidence Interval with a type I

error of 5%.
