# Supplementary material for: Income level and regional policies, underlying factors associated with unwarranted variations in conservative breast cancer surgery in Spain
Source: BMC Cancer. 2011 Apr 19;11:145. doi: 10.1186/1471-2407-11-145 (PMC3103476; doi:10.1186/1471-2407-11-145)
Supplement: Additional file 3 — Joint point analysis of change in utilisation trends for conservative and non conservative surgery per age group. The file illustrates the joint-model estimates, testing whether the early adoption of conservative surgery explained part of the variation. [file 1471-2407-11-145-S3.DOC]

**Additional file 3**

Joint point analysis of change in utilisation trends for conservative and non conservative surgery per age group

| **1. All ages.** | | | | | | Joint Point Regression | | | | | |
| --- | --- | --- | --- | --- | --- | --- | --- | --- | --- | --- | --- |
|  |  |  |  |  |  |  | Trend 1 | | Trend 2 | |  |
| Years | 2002 | 2003 | 2004 | 2005 | 2006 | PAG 2002-2006 | Period | PAG | Period | PAG | P-value |
| CS Cases | 7833 | 8361 | 8714 | 9510 | 10230 |  |  |  |  |  |  |
| CS Standardized rate | 4.81 | 5.01 | 5.11 | 5.49 | 5.98 | 5.4 | 2002-2004 | 2.9 | 2004-2006 | 8 | 0.022 |
| NCS Cases | 7419 | 7767 | 7629 | 7631 | 7621 |  |  |  |  |  |  |
| NCS Standardized rate | 4.87 | 5.04 | 4.95 | 4.72 | 4.62 | - 1.65 | - - | - - | - - | - - | 0.124 |
| Population | 15841380 | 16106664 | 16268660 | 16565336 | 16824239 |  |  |  |  |  |  |
| **2. Less than 50 years old.** | | | | | | Joint Point Regression | | | | | |
|  |  |  |  |  |  |  | Trend 1 | | Trend 2 | |  |
| Years | 2002 | 2003 | 2004 | 2005 | 2006 | PAG 2002-2006 | Period | PAG | Period | PAG | P-value |
| CS Cases | 2047 | 2197 | 2373 | 2475 | 2729 |  |  |  |  |  |  |
| CS Standardized rate | 2.13 | 2.22 | 2.40 | 2.55 | 2.72 | 6.6 | - - | - - | - - | - - | 0.837 |
| NCS Cases | 1810 | 1892 | 1932 | 1944 | 2068 |  |  |  |  |  |  |
| NCS Standardized rate | 1.93 | 2.02 | 2.09 | 2.05 | 2.13 | 2.20 | - - | - - | - - | - - | 0.291 |
| Population | 9292284 | 9438314 | 9516511 | 9680873 | 9800845 |  |  |  |  |  |  |
| **3. 50 to 70 years old.** | | | | | | Joint Point Regression | | | | | |
|  |  |  |  |  |  |  | Trend 1 | | Trend 2 | |  |
| Years | 2002 | 2003 | 2004 | 2005 | 2006 | PAG 2002-2006 | Period | PAG | Period | PAG | P-value |
| CS Cases | 4169 | 4354 | 4554 | 4873 | 5254 |  |  |  |  |  |  |
| CS Standardized rate | 10.56 | 10.70 | 10.64 | 11.47 | 12.24 | 3.7 | 2002-2004 | 0.39 | 2004-2006 | 7.07 | 0.007 |
| NCS Cases | 2988 | 3066 | 2981 | 2798 | 2843 |  |  |  |  |  |  |
| NCS Standardized rate | 8.04 | 8.07 | 7.96 | 7.17 | 7.31 | - 3.03 | - - | - - | - - | - - | 0.608 |
| Population | 3891438 | 3940353 | 3966603 | 4030879 | 4102445 |  |  |  |  |  |  |
| **4. More than 70 years old.** | | | | | | Joint Point Regression | | | | | |
|  |  |  |  |  |  |  | Trend 1 | | Trend 2 | |  |
| Years | 2002 | 2003 | 2004 | 2005 | 2006 | PAG 2002-2006 | Period | PAG | Period | PAG | P-value |
| CS Cases | 1617 | 1810 | 1787 | 2162 | 2247 |  |  |  |  |  |  |
| CS Standardized rate | 5.77 | 6.47 | 6.50 | 7.03 | 8.12 | 7.7 | - - | - - | - - | - - | 0.463 |
| NCS Cases | 2621 | 2809 | 2716 | 2889 | 2710 |  |  |  |  |  |  |
| NCS Standardized rate | 10.53 | 11.12 | 10.44 | 10.29 | 9.19 | - 3.28 | - - | - - | - - | - - | 0.191 |
| Population | 2657658 | 2727997 | 2785546 | 2853584 | 2920949 |  |  |  |  |  |  |

CS Conservative Surgery; NCS: Non conservative Surgery. PAG: Percentage of annual growth in rates
